# Identification of a molecular gating determinant within the carboxy terminal region of Ca_v_3.3 T-type channels

**DOI:** 10.1186/s13041-019-0457-0

**Published:** 2019-04-08

**Authors:** Bohumila Jurkovicova-Tarabova, Leos Cmarko, Renata Rehak, Gerald W. Zamponi, Lubica Lacinova, Norbert Weiss

**Affiliations:** 1 Center of Biosciences, Institute of Molecular Physiology and Genetics, Academy of Sciences, Bratislava, Slovakia; 2 Institute of Organic Chemistry and Biochemistry, Czech Academy of Sciences, Flemingovo nam. 2, 16610 Prague, Czech Republic; 3 Department of Physiology and Pharmacology, Cumming School of Medicine, University of Calgary, Calgary, Canada

**Keywords:** T-type channels, Ca_v_3.3 channel, Gating, Electrophysiology

## Abstract

The physiological functions controlled by T-type channels are intrinsically dependent on their gating properties, and alteration of T-type channel activity is linked to several human disorders. Therefore, it is essential to develop a clear understanding of the structural determinants responsible for the unique gating features of T-type channels. Here, we have investigated the specific role of the carboxy terminal region by creating a series a deletion constructs expressed in tsA-201 cells and analyzing them by patch clamp electrophysiology. Our data reveal that the proximal region of the carboxy terminus contains a structural determinant essential for shaping several gating aspects of Ca_v_3.3 channels, including voltage-dependence of activation and inactivation, inactivation kinetics, and coupling between the voltage sensing and the pore opening of the channel. Altogether, our data are consistent with a model in which the carboxy terminus stabilizes the channel in a closed state.

## Introduction

Low voltage-activated T-type calcium channels support essential functions both in excitable and nonexcitable cells [1]. The molecular cloning of T-type channels has revealed the existence of three distinct channel isoforms, namely Ca_v_3.1 [2], Ca_v_3.2 [3], and Ca_v_3.3 [4]. Ca_v_3.1 and Ca_v_3.2 are rather ubiquitously expressed and are found in the nervous system, neuroendocrine cells, cardiovascular system, and even the reproductive system [1]. In contrast, Ca_v_3.3 channels are mostly expressed in the nervous system, although the presence of the channel in neuroendocrine tissues has been suggested. In the brain, Ca_v_3.3 channels are highly expressed in the olfactory bulb, cortex, midbrain/diencephalon, and in the cerebellum, and to a lesser extent in the hippocampus and pons/medulla [5–7]. In addition, Ca_v_3.3 channels were documented in rat pancreatic beta cells [8], human spermatogenic cells [9], and in interstitial cells of Cajal in rat bladder [10].

T-type channels operate at negative voltages near the resting membrane potential of nerve cells [1], where they contribute to neuronal excitability by generating low-threshold calcium spikes and action potential bursts [11] that are implicated in both normal and pathological conditions [12]. In addition, the overlap between their voltage-dependence of activation creates a window current for calcium influx at rest, which contributes to the bistability of the membrane potential [13] and is essential for the generation of slow neuronal oscillations that occur during sleep [14]. Therefore, the contribution of T-type channels in neuronal excitability relies on the specific voltage-dependent gating properties of the channels. Several studies using a chimeric approach, which consists of swapping structural regions between channel isoforms, have identified a number of regions that are important for voltage-dependent gating of T-type channels. These structural determinants span across the channel protein and include the transmembrane domains I and IV, as well as several cytoplasmic regions including the I-II loop, III-IV loop, and the carboxy terminal region [15–19]. However, the chimeric approach does not allow pinpointing detailed channel structural determinants of gating, especially if these determinants are present within different channel isoforms.

In this study, we have analyzed the role of the carboxy (C)-terminal region of Ca_v_3.3 in the gating of the channel by using a deletion approach. We demonstrate that the initial proximal region of the C-terminus contains a stretch of 20 amino acids that is highly conserved among the three T-type channel isoforms and essential for setting the gating of Ca_v_3.3 channels. Removal of this region produced a robust shift in the voltage-dependence of activation and inactivation of the channel toward more hyperpolarized potentials, slowed channel inactivation, and decreased the efficiency of the coupling between the voltage sensing and the pore opening of the channel. Remarkably, all of these alterations were fully restored upon coexpression of a plasma membrane anchored fusion protein containing the proximal C-terminal region of Ca_v_3.2. Altogether, our findings uncover a unique role of a discreet structural determinant in setting voltage-dependent gating of Ca_v_3.3 channels.

## Results

### The carboxy terminus of Ca_v_3.3 modulates channel activation

**Fig. 1:**
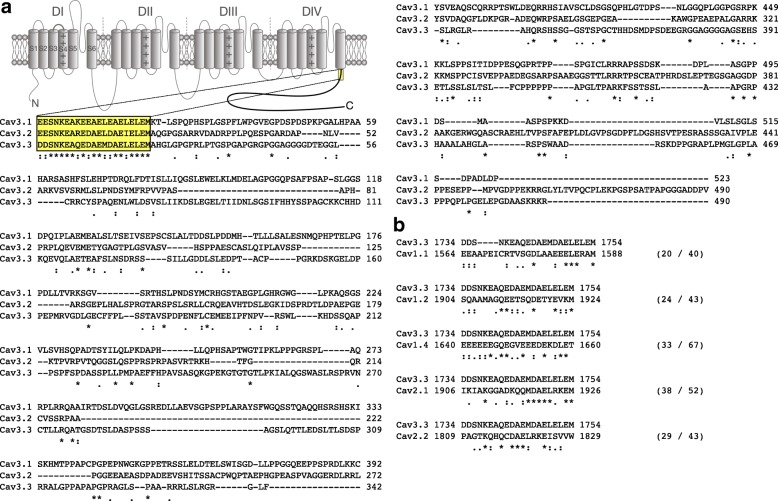
Identification of a conserved region within the carboxyl terminal domain of T-type channels. **a** Schematic representation of the T-type channel membrane topology and protein sequence alignment of the carboxyl terminal domain of the three T-type channel isoforms (Ca_v_3.1, Ca_v_3.2, and Ca_v_3.3) showing a stretch of highly conserved residues within the proximal region (highlighted in yellow). **b** Protein sequence alignment of the conserved proximal C-terminal region of Ca_v_3.3 with the C-terminal domain of high-voltage-activated (HVA) calcium channels. Only HVA channels for which some degree of homology exist are shown. Numbers in bracket indicate the percentage of identity and similarity, respectively

**Fig. 2:**
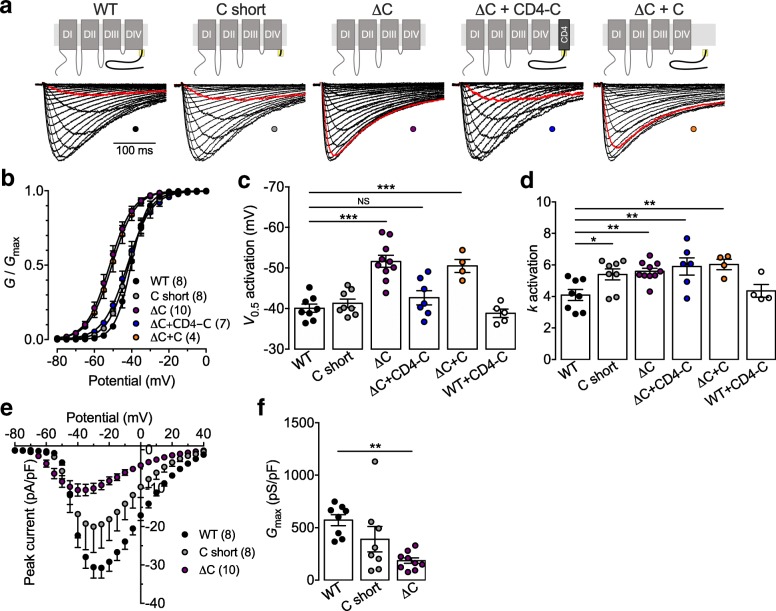
Role of C-terminus on Ca_v_3.3 activation. **a** Representative set of current traces recorded in response to 300 ms depolarizing steps ranging from − 80 mV to 0 mV from a holding potential of − 100 mV from cells expressing the different Ca_v_3.3 channel combinations indicated above the current traces. The red trace indicates the current recorded in response to a depolarization step at − 50 mV. **b** Corresponding mean normalized voltage dependence of activation. **c** Corresponding mean half activation potential. **d** Corresponding mean activation slope factor. **e** Current-voltage relationships for cells expressing WT (black circles), C short (grey circles), and ΔC (purple circles) channel constructs. **f** Corresponding mean maximal conductance

While the C-terminal domain of T-type channels shares 7% identity only between the three channel isoforms (Ca_v_3.1, Ca_v_3.2, and Ca_v_3.3), the proximal region contains a highly conserved stretch of 21 residues with 67% identity and 100% similarity between the channel isoforms, suggesting a potentially important role of this region in the functioning of the channel (Fig. 1a). In contrast, this molecular structure appears with only a weak degree of homology in several high-voltage-gated calcium channel isoforms and much downstream of the proximal region of the carboxy terminus (Fig. 1b). To investigate the role of this conserved region in the gating of Ca_v_3.3, we generated deletion mutants of Ca_v_3.3 channels where the C-terminal region was either entirely removed (ΔC) or where the conserved proximal region was preserved (C short). Representative whole-cell T-type currents recorded from tsA-201 cells expressing Ca_v_3.3 deletion constructs in response to 300 ms depolarizing steps to values ranging between − 90 mV and + 30 mV from a holding potential of − 100 mV are shown in Fig. 2a. The mean normalized voltage-dependence of activation is shown in Fig. 2b. While the mean half activation potential of the C short variant was virtually identical (*p* = 0.9494) to that of the WT channel (− 41.3 ± 1.1 mV, *n* = 8; versus − 40.1 ± 1.0, *n* = 8), deletion of the entire carboxyl terminal region (ΔC) induced a shift of 11.5 mV (*p* < 0.0001) toward more hyperpolarized potentials (− 51.6 ± 1.5 mV, *n* = 10) (Fig. 2b and c). Remarkably, coexpression of the membrane anchored CD4-tagged C-terminus of Ca_v_3.2 (CD4-C) was able to fully rescue the voltage-dependence of activation of the ΔC channel variant (− 42.7 ± 1.7 mV, *n* = 7), without affecting the properties of the WT channel (Fig. 2b and c). In contrast, rescue of the ΔC channel variant was not observed upon expression of the cytosoluble form of the Ca_v_3.2 C-terminus (C), indicating that membrane anchoring of the C-terminus is essential for modulating the gating of the channel (Fig. 2b and c). Importantly, the observation that the CD4-C construct was able to rescue the gating of the ΔC variant indicates that alteration of Ca_v_3.3 channel activation upon deletion of the entire C-terminus is not a consequence of a faulty embedding of the channel in the plasma membrane, but rather the result of the deletion of the gating molecular determinant within the C-terminal region of the channel. Additionally, a minor but significant increase in the activation slope factor was observed upon deletion of the C-terminus (partially and entirely), which was not rescued by coexpression of the CD4-C (Fig. 2d). In addition, the maximal conductance of cells expressing the Ca_v_3.3 ΔC variant was decreased by 67% (*p* = 0.0011) compared to cells expressing the WT channel (from 572 ± 53 pS/pF, *n* = 8 to 186 ± 26 pS/pF, *n* = 10) (Fig. 2e and f). The effect was rescued by the co-expression of the CD4-C construct (204 ± 38 pS/pF, *n* = 7). In contrast, the maximal conductance of cells expressing the C short construct (390 ± 121 pS/pF, *n* = 8) was not significantly different (*p* = 0.1888) compared to cells expressing the WT channel. These results suggest that in addition to control the gating of Ca_v_3.3, the proximal Cter region may also influence the expression and/or trafficking of the channel to the plasma membrane.

Altogether, these data indicate that the conserved proximal region of the carboxyl terminal modulates the voltage-dependence of activation of Ca_v_3.3 and acts as a molecular brake to prevent the channel to open at more hyperpolarized potentials.

### The carboxy terminus of Ca_v_3.3 modulates channel inactivation

**Fig. 3:**
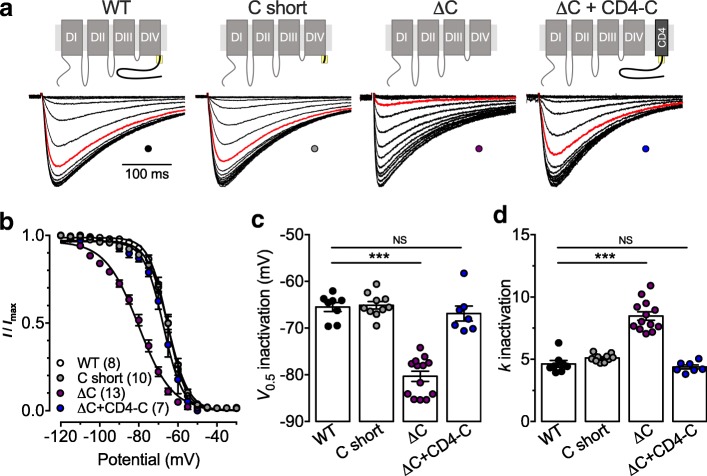
Role of C-terminus on Ca_v_3.3 inactivation **a** Representative set of current traces recorded in response to 300 ms depolarizing step to − 20 mV from a holding potential of − 100 mV, preceded by 15 s inactivating pulse ranging from − 120 mV to − 30 mV from cells expressing the different Ca_v_3.3 channel combinations indicated above the current traces. The red trace indicates the current recorded after an inactivating pulse at − 60 mV. **b** Corresponding mean normalized voltage dependence of steady state inactivation. **c** Corresponding mean half inactivation potential. **d** Corresponding mean steady state inactivation slope factor

We further investigated the role of the C-terminal region on the inactivation properties of Ca_v_3.3 channels. The steady state inactivation (SSI) of Ca_v_3.3 deletion mutants was analyzed using a double pulse protocol (see Methods). Representative current traces in response to a 300 ms depolarizing step to 0 mV preceded by a 15 s inactivating pulse ranging from − 120 mV to − 30 mV are shown in Fig. 3a. The mean-normalized voltage-dependence of SSI is shown in Fig. 3b for WT, C short, and ΔC channels. Similar to what we observed with the voltage-dependence of activation, the mean half-SSI potential was shifted toward more negative potentials by 14.9 mV (*p* < 0.0001) in cells expressing the ΔC variant (− 80.3 ± 1.1 mV, *n* = 13) compared to cells expressing the WT channel (− 65.5 ± 1.0 mV, *n* = 8) (Fig. 3c). In contrast, the mean half-SSI potential of the C short variant remained unaltered (− 65.1 ± 0.8 mV, *n* = 10; *p* = 0.9922). Additionally, coexpression of the CD4-C construct was able to restore the voltage-dependence of inactivation of the ΔC variant to values indistinguishable from control values (− 66.9 ± 1.6 mV, *n* = 7; *p* = 0.7856) (Fig. 3c). We also observed a 1.8-fold increase (*p* < 0.0001) in the inactivation slope factor of the voltage-dependence of inactivation of the ΔC variant (8.5 ± 0.3, *n* = 13) compared to WT channels (4.6 ± 0.3, *n* = 8), which was restored upon coexpression of the CD4-C construct (4.4 ± 0.2, *n* = 7; *p* = 0.8885) (Fig. 3c).

**Fig. 4:**
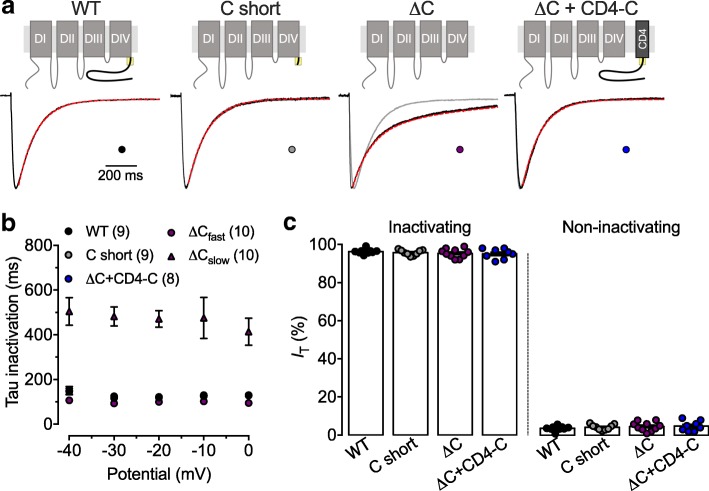
Role of C-terminus on Ca_v_3.3 inactivation kinetics. **a** Representative current traces recorded in response to 1 s depolarizing step to − 20 mV from a holding potential of − 100 mV from cells expressing the different Ca_v_3.3 channel combinations indicated above. The dotted red line indicates the fit of the current decay by a single (WT, C short, and ΔC + CD4-C) or double exponential function (ΔC). The grey line represents the WT current for comparison. **b** Corresponding mean time constant of inactivation. **c** Corresponding mean fractions of inactivating and non-inactivating current

The inactivation kinetics were analyzed by fitting the decay phase of the T-type current with an exponential function (Fig. 4a). Representative current traces in response to a 1 s depolarization step to − 20 mV from a holding potential of − 110 mV are shown in Fig. 4. Inactivation of the WT and C short channels were perfectly fitted with a single exponential function (Fig. 4a). For instance, the time constant of inactivation of the C short channel at − 20 mV (122 ± 6 ms, *n* = 9) was similar (*p* = 0.8911) to that of the WT channel (119 ± 4 ms, *n* = 9) (Fig. 4b). In contrast, inactivation of the ΔC variant was best fitted with a double exponential function (the correlation coefficient increased from 0.9368 ± 0.007 to 0.9564 ± 0.007) (Fig. 4a) with a fast time constant significantly faster (*p* = 0.0239) than what was observed for WT channels (100 ± 7 ms, *n* = 10), and an additional slow component (471 ± 37 ms, *n* = 10) (Fig. 4b). While the fast inactivation component was responsible for 38 ± 6% of total current, deletion of the C-terminus had no effect on the total fraction of inactivating current (Fig. 4c). Coexpression of the CD4-C construct was able to restore inactivation properties of the Ca_v_3.3 ΔC channel to a single component (Fig. 4a and b).

**Fig. 5:**
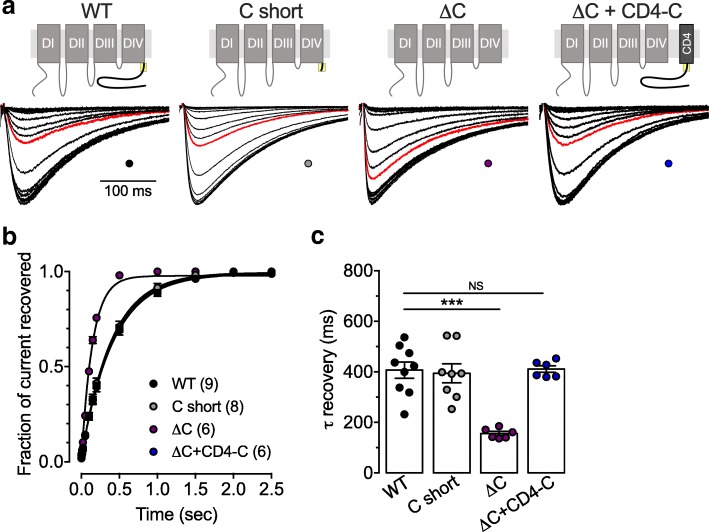
Role of C-terminus on Ca_v_3.3 recovery from inactivation. **a** Representative current traces recorded in response to a 300 ms depolarizing step to − 20 mV preceded by a recovery pulse at − 110 mV for a duration ranging between 0.1 ms and 2500 ms after a 15 s period of inactivation at 0 mV from cells expressing the different Ca_v_3.3 channel combinations indicated above the current traces. The red line represents the current recorded after 200 ms recovery. **b** Corresponding mean normalized recovery from inactivation. **c** Corresponding mean time constant of recovery from inactivation

Finally, we analyzed recovery from inactivation (RFI). Representative current traces in response to a 300 ms depolarizing step to − 20 mV preceded by a recovery pulse at − 110 mV for a duration ranging between 0.1 ms and 2500 ms after a 15 s period of inactivation at 0 mV are shown in Fig. 5a. The mean-normalized current recovery is shown in Fig. 5b for WT, C short, and ΔC channel variants. Deletion of the C-terminus significantly accelerated (*p* < 0.0001) the time constant of RFI (156 ± 8, *n* = 6) compared to WT channels (407 ± 32, *n* = 9) (Fig. 5c). In contrast, RFI of the C short channel variant remained unaffected (394 ± 37, *n* = 8; *p* = 0.7979) (Fig. 5c). Similar to what we observed for the voltage-dependence of activation and inactivation, RFI of the Ca_v_3.3 ΔC channels was fully restored upon coexpression of the CD4-C construct (Fig. 5b and c).

Taken together, these data indicate that the conserved proximal region of the Ca_v_3.3 C-terminus plays a complex role in the control of the inactivation properties of Ca_v_3.3 channels. On one hand it prevents steady state inactivation of the channel, and on the other hand it potentiates fast inactivation, but also slows down recovery from inactivation.

### The carboxy terminal region of Ca_v_3.3 modulates the window current

**Fig. 6:**
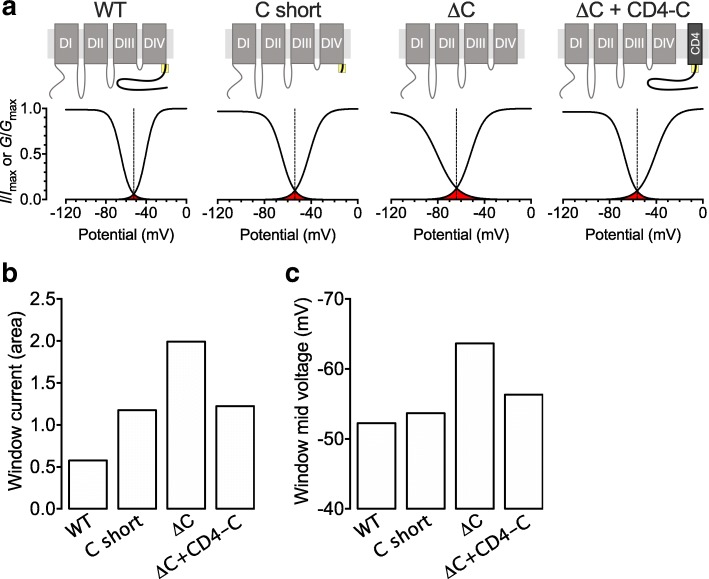
Role of C-terminus on Ca_v_3.3 window current. **a** Window current (red) obtained from the mean activation and inactivation curves for Ca_v_3.3 channel combination indicated above. **b** Amplitude of the window current measured as the area of the overlap between the activation and inactivation curves. **c** Mid voltage of the window current measured at the intersection between the activation and inactivation curves

Considering that deletion of the C-terminus alters both the voltage-dependences of activation and inactivation, we analyzed the role of the C-terminus in setting the window current supported by Ca_v_3.3 channels. The window current is presented in Fig. 6a as the area under the overlap between the activation and inactivation curves for each channel variant. Upon partial deletion of the C-terminus (C short), the window current was increased by 2-fold, while deletion of the entire C-terminus (ΔC) increased the window current by 3.5-fold compared to WT channels (Fig. 6b). Consistent with the observation that the CD4-C construct was not able to restore the activation slope of the C-terminal deletion variants (both C short and ΔC), coexpression of CD4-C with Ca_v_3.3 ΔC channels only restored the window current to the level of Ca_v_3.3 C short (Fig. 6b). Additionally, the mid voltage of the window current was shifted by 11.4 mV toward hyperpolarized potentials upon deletion of the entire C-terminus (Fig. 6c).

These data indicated that the conserved C-terminal region, by modulating the voltage properties of Ca_v_3.3, also influences the amplitude and voltage of the channel’s window current.

### The carboxyl terminus of Ca_v_3.3 contributes to the coupling between voltage sensing and the channel opening

**Fig. 7:**
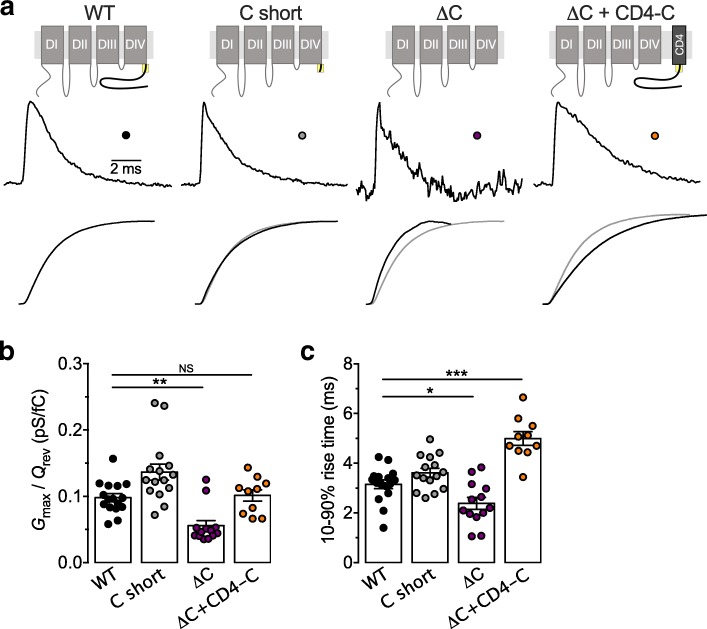
Role of C-terminus on Ca_v_3.3 gating currents. **a** Representative gating current traces recorded from cells expressing the various Ca_v_3.3 variants. Each trace represents an average of five traces recorded during cell depolarization to its reversal potential. Below each current trace is a depiction of time courses of integrals calculated for that trace. Current traces and integrals are scaled to 1 for a better comparison. Grey lines represent the integral of the gating current trace for the WT channel. **b** Corresponding mean G_max_/Q_rev_ ratios calculated for each investigated cell. **c** Corresponding mean 10–90% rise times calculated from the integral time course shown in panel **a**

The hyperpolarized shift of the voltage-dependence of the channel gating upon deletion of the C-terminus may have resulted from the alteration of the effectiveness of coupling between the activation of the voltage sensor and the opening of the pore of the channel. Therefore, we performed a set of experiments to assess the gating currents of Ca_v_3.3 channels. Gating currents were measured at the reversal potential of the T-type current where there is no contamination from ionic current [20]. Representative gating current traces recorded from HEK-293 cells expressing Ca_v_3.3 deletion variants are shown in Fig. 7a. Gating charge Q_rev_ from Ca_v_3.3 channels measured at the reversal potential can be considered to closely approximate the maximal gating charge [21]. The maximal conductance *G*_max_ for each investigated cell was calculated by fitting experimental data of the current-voltage relationship with equation [1]. The relation between the maximal conductance G_max_ and gating charge Q_rev_ characterizes the coupling between the activation of the voltage sensor and the pore opening of the channel [18, 20]. Upon deletion of the entire C-terminus (ΔC), we observed a significant decrease (*p* = 0.0148) in the *G*_max_/*Q*_rev_ ratio (0.056 ± 0.008 pS/fC, *n* = 13) compared to WT channels (0.098 ± 0.006 pS/fC, *n* = 16) (Fig. 7b). In contrast, *G*_max_/*Q*_rev_ ratio of the C short channel variant remained unaffected (0.137 ± 0.012 pS/fC, *n* = 15, *p* = 0.0597) (Fig. 7b). Additionally, deletion of the entire C-terminus significantly accelerated (*p* < 0.0287) the kinetics of charge movements (2.39 ± 0.23 ms, *n* = 13) compared to WT channels (3.15 ± 0.17 ms, *n* = 13) (Fig. 7c). Coexpression of the CD4-C construct was able to fully restore *G*_max_/*Q*_rev_ ratio of Ca_v_3.3 ΔC channels to values not significantly different from WT values (*p* > 0.9999) (Fig. 7b), and the kinetics of charge movements were not only restored but also significantly slowed (4.99 ± 0.28 ms, *n* = 10, *p* < 0.0001) (Fig. 7c).

Collectively, these data indicate that the proximal region of the C-terminus of Ca_v_3.3 contributes to the coupling between the channel voltage sensor and pore opening.

## Discussion

Voltage-dependent gating of T-type channels is an essential determinant of physiological functions supported by these channels. Hence, fast inactivating Ca_v_3.1 and Ca_v_3.2 channels support the burst-firing mode of action potentials in thalamic neurons [11], while the comparatively slowly inactivating Ca_v_3.3 channels mediate pacemaker activity [22]. Previous studies using a chimeric approach have identified several channel regions that contribute to the unique gating properties of T-type channels. For instance, Hamid and colleagues have revealed that the transmembrane domain IV region is essential for T-type channel activation, while multiple structural determinants contribute to channel inactivation [16]. Using a similar approach, several additional studies have pointed to a role of the domain I region, and of cytoplasmic I-II and III-IV loops in T-type channel activation and inactivation properties [17–19]. Although such a chimeric approach is a powerful approach for identifying the structural determinants responsible for differences in specific gating properties between channel isoforms, it does not allow one to pinpoint detailed structural determinant of gating when these determinants are conserved within different channel isoforms. In this study, we have investigated the specific contribution of the C-terminal domain of Ca_v_3.3 to the gating of the channel. We provide evidence that the proximal region of the C-terminus contains a stretch of 20 residues that is essential for the voltage-dependent gating of Ca_v_3.3 channels.

The C-terminal region of voltage-gated calcium channels is implicated in several regulatory aspects including modulation by calcium, phosphorylation, and synaptic proteins [23–26]. Here, we demonstrate that the C-terminus of Ca_v_3.3 is an essential structural determinant that contributes to several gating aspects of the channel. We show that deletion of the entire C-terminus (Ca_v_3.3 ΔC), leads to a dramatic shift of the voltage-dependences of activation and inaction of the channel towards more hyperpolarized potentials. In addition, we observed several alterations in the kinetics of the channel including reduced channel inactivation and faster recovery from inactivation. Finally, recordings of gating charge movement revealed a decreased coupling between the voltage sensing and pore opening of the channel. Although these alterations could have resulted from a nonspecific alteration of the channel protein, our observation that most of the gating properties were fully restored upon coexpression of a membrane anchored fusion protein containing the C-terminus of Ca_v_3.2 channel indicates that alteration of Ca_v_3.3 gating is an intrinsic consequence of the deletion of C-terminal region rather than a nonspecific misfolding of the channel. In addition, the Ca_v_3.3 C short deletion channel (where the entire C-terminus except for the first 20 proximal amino acids) retained gating properties that were undistinguishable from the full-length channel. This suggests that alteration of Ca_v_3.3 gating upon deletion of the C-terminus is essentially mediated by the absence of this proximal structural determinant. This notion is further supported by the observation that despite the fact that the C-terminus of Ca_v_3.2 presents a very low degree of homology with the C-terminus of Ca_v_3.3 (14% identity and 23% similarity) and is only highly homologue for the first 21 amino acids (76% identity and 90% similarity), it was sufficient to rescue the gating of Ca_v_3.3 ΔC, further excluding the implication of downstream structural determinants.

Most of the gating effects observed in this study can be explained by the notion that the proximal C-terminal region of Ca_v_3.3 modulates channel gating by stabilizing the channel in a closed state. This notion is consistent with our observation that the voltage-dependence of activation is shifted toward more hyperpolarized potentials upon deletion of the C-terminus. In addition, T-type channels inactivate from both open and closed states, and inactivation from the closed state is especially prominent for Ca_v_3.3 channels [27, 28]. Considering that activation and inactivation of T-type channels are allosterically coupled [27, 28], the voltage-dependence of channel inactivation would also be shifted toward more hyperpolarized potentials upon removal of the C-terminus. In addition, we note the emergence of an additional slow current inactivation component that is observed in the absence of the C-terminus, without alteration of the maximal extent of voltage-dependent inactivation. Finally, the inactivated state reached form the open state may be less stable, which may have contributed to the faster recovery from inactivation observed in the absence of the C-terminus. This model is further supported by our observation that the kinetics of gating currents were accelerated in the absence of the C-terminus. Considering that the C-terminus of Ca_v_3.3 is unlikely to affect the unitary conductance of the channel, our observation that G_max_/Q_rev_ was decreased upon removal of the C-terminus suggests a decreased opening probability of the channel, which may be explained by increased channel inactivation from the open state.

There are different ways by which deletion of the C-terminus may have affected the gating of Ca_v_3.3 channels. For instance, it was recently reported that the C-terminus of Ca_v_3.3 contributes to calcium-dependent inactivation of the channel [29]. This regulation requires the binding of calmodulin within the C-terminal region of the channel and is limited to Ca_v_3.3 channels. However, it is unlikely that such a modulation may have contributed to the effects that we observed in this study. First, alteration of Ca_v_3.3 gating upon deletion of the C-terminus was observed in the absence of extracellular calcium and therefore a calcium-dependent regulation of the channel could not have occurred. And second, we demonstrated that the observed gating effects rely on a short proximal region of the C-terminus, which does not contain canonical calmodulin binding motifs. In contrast, it is a possibility that this region mediates its effects by interacting with additional regulatory proteins, or directly within the channel itself. Further investigation is necessary to reveal the detailed molecular mechanism by which the C-terminus contributes to the gating the channel and possibly unravel intramolecular interactions within the channel protein.

## Methods

### Plasmid cDNA constructs

Human Ca_v_3.3 wild type in pMT2 vector was previously described [16] and used as a template to generate the carboxy terminal deletion variants. Ca_v_3.3 ΔC was generated by PCR using a 5′ primer made 900 bp upstream of the start of the carboxy termimus and a 3′ primer containing a stop codon made at the end of the IV6 transmembrane segment. The PCR product was cloned into pGEMTeasy and subclone into the full length Ca_v_3.3 into BmgBI and XhoI. For generation of the Ca_v_3.3 C short, the pMT2-Ca_v_3.3 clone was cut with XhoI and religated. This left the first 20 proximal residues of the carboxyl terminus and a stop codon in the pMT2 vector. To generate the CD4-taged Ca_v_3.2 Cter construct, the carboxyl terminus of the human Ca_v_3.2 (amino acid 1864–2353) was amplified by PCR. The PCR product was cloned into the hCD4-mOrange plasmid (addgene) and the CD4-Ca_v_3.2 Cter was cut and subcloned into the pcDNA3.1 vector. All constructs were verified by sequencing.

### Heterologous expression

Human embryonic kidney tsA-201 cells were grown in DMEM medium supplemented with 10% fetal bovine serum (FBS) and 1% streptomycin/penicillin (all media from Invitrogen), and maintained under standard conditions at 37 °C in a humidified atmosphere containing 5% CO_2_. Cells were plated out onto 60 mm dishes and transfected using the calcium/phosphate method with cDNAs encoding for the respective human Ca_v_3.3 channel constructs.

### Patch-clamp electrophysiology

Patch-clamp recordings were performed 72 h after transfection in the whole-cell configuration of the patch-clamp technique at room temperature (22–24 °C) in a bath solution containing (in millimolar): 5 BaCl2, 5 KCl, 1 MgCl2, 128 NaCl, 10 TEA-Cl, 10 D-glucose, 10 4-(2-hydroxyethyl)-1-piperazineethanesulfonic acid (HEPES) (pH 7.2 with NaOH). Patch pipettes had a resistance of 2–4 MΩ when filled with a solution containing (in millimolar): 110 CsCl, 3 Mg-ATP, 0.5 Na-GTP, 2.5 MgCl2, 5 D-glucose, 10 EGTA, and 10 HEPES (pH 7.4 with CsOH). Whole-cell patch-clamp recordings were performed using an Axopatch 200B amplifier (Axon Instruments). Acquisition and analysis were performed using pClamp 10 and Clampfit 10 software, respectively (Axon Instruments).

Ba^2+^ currents were recorded in response to 300 ms or 1 s depolarizing steps to various potentials applied every 10 s from a holding potential of − 100 mV. The linear leak component of the current was corrected online and current traces were digitized at 10 kHz, and filtered at 2 kHz. The voltage dependence of the peak Ba^2+^ current was fitted with the following modified Boltzmann equation (1):

1 $$ I(V)= Gmax\ \frac{\left(V-V\mathrm{rev}\right)}{1+\exp \frac{\left(V0.5-V\right)\ }{k}} $$

with *I*(*V*) being the peak current amplitude at the command potential *V*, *G*max the maximum conductance, *V*rev the reversal potential, *V*_0.5_ the half-activation potential, and *k* the slope factor. The voltage dependence of the whole-cell Ba^2+^ conductance was calculated using the following modified Boltzmann equation (2):

2 $$ G(V)=\frac{Gmax}{1+\exp \frac{\left(V0.5-V\right)}{k}} $$

with *G*(*V*) being the Ba^2+^ conductance at the command potential *V*.

The steady state voltage-dependence of inactivation of the Ba^2+^ current was determined by measuring the peak current amplitude in response to a 300 ms depolarizing step to − 20 mV applied after a 15 s conditioning prepulse ranging from − 120 mV to − 30 mV. The current amplitude obtained during each test pulse was normalized to the maximum at − 120 mV and plotted as a function of the prepulse potential. The voltage-dependence of the steady state inactivation was fitted with the following two-state Boltzmann function (3):

3 $$ I(V)=\frac{Imax}{1+\exp \frac{\left(V-V0.5\right)\ }{k}} $$

with *I*_max_ corresponding to the maximal peak current amplitude and *V*_0.5_ to the half-inactivation voltage.

The recovery from inactivation was studied using a double-pulse protocol from a holding potential of − 110 mV. The cell membrane was depolarized for 15 s to 0 mV (prepulse) to ensure complete inactivation of the channel and then to − 20 mV for 300 ms (test pulse) after an increasing time period (interpulse interval) between 0.1 ms and 2 s at − 110 mV. The peak current from the test pulse was plotted as a ratio of the maximum prepulse current versus interpulse interval. The data were fitted with a single-exponential function (4):

4 $$ \frac{I}{Imax}=A\times \left(1-\mathit{\exp}\frac{-t}{\tau}\right) $$

where τ the time constant of the exponential component.

### Measurement of gating currents

HEK 293 cells were cultured in MEM supplemented with Earl’s salts, 10% bovine calf serum and 1% penicillin/streptomycin and maintained under regular conditions. Cells were transfected using jetPRIME transfection reagent (Polyplus transfection). Gating currents were measured 72 h after transfection at room temperature in a bath solution containing (in millimolar): CsCl 95; TEACl 40, BaCl_2_ 5; MgCl_2_ 1; HEPES 10; glucose 10; pH 7.4 (adjusted with CsOH). Patch pipettes had a resistance ranging from 1.8 MΩ to 2.2 MΩ when filled with a solution containing (in millimolar): CH_3_SO_3_Cs 130; Na-ATP 5; TEACl 10; HEPES 10; EGTA 10; MgCl_2_ 5; pH 7.4 (adjusted with CsOH). Osmolarity of the intracellular solution was approximately 300 mOsmol/L. Osmolarity of the extracellular solution was adjusted by adding sucrose so that the final value was about 2–3 mOsmol/L lower than the osmolarity of the corresponding intracellular solution. Recordings were performed using HEKA EPC10 amplifier (HEKA Electronics). Acquisition and analysis were performed using Patchmaster v90.2 and Fitmaster v2x73.1 and Origin Pro 2015 software, respectively. Only cells with an input resistance less than 5 MΩ were considered. The input resistance and capacity transients were compensated with in-built circuits of the EPC 10 amplifier up to 70%. Remaining artifacts were subtracted using a -P/8 procedure. For each cell, current/voltage relationship was measured by a series of 50 ms long depolarizing pulses applied every 5 s from a holding potential of − 100 mV to voltages between − 100 mV and + 70 mV with + 10 mV increment. Experimental data for each cell were fitted with the modified Boltzmann-Ohm equation (1). Actual reversal potential was measured for each investigated cell and ON-gating currents were measured by a series of 5 depolarizing pulses at the reversal potential. Recorded gating current traces were averaged and total gating charge Q_ON_ was calculated as the integral of area below the averaged current trace. The kinetics of channel gating were evaluated from the time course of gating current integral as a 10–90% rise time.

### Statistical analysis

Data values are presented as mean ± S.E.M. for *n* experiments. Statistical significance was determined using a one-way ANOVA test followed by Dunnett multiple comparison test. * *p* < 0.05, ** *p* < 0.005, *** *p* < 0.001.

## Abbreviations

C short: Ca_v_3.3 channel deleted of the entire C-terminus except for the first 20 proximal amino acids
CD4-C: CD4-tagged C-terminus of Ca_v_3.2
G_max_: Maximal macroscopic conductance
Q_rev_: Gating charge at the reversal calcium potential
WT: Wild type
ΔC: Ca_v_3.3 channel deleted of the entire C-terminus
